# Changes in Attitudes of Japanese Doctors toward Complementary and Alternative Medicine—Comparison of Surveys in 1999 and 2005 in Kyoto

**DOI:** 10.1093/ecam/nep040

**Published:** 2011-06-23

**Authors:** Kenji Fujiwara, Jiro Imanishi, Satoko Watanabe, Kotaro Ozasa, Kumi Sakurada

**Affiliations:** ^1^Department of Microbiology, Kyoto Prefectural University of Medicine, Kawaramachi-Hirokoji, Kamikyo-ku, Kyoto 602-8566, Japan; ^2^Department of Social Medicine and Cultural Sciences, Research Institute for Neurological Diseases and Geriatrics, Kyoto Prefectural University of Medicine, Kawaramachi-Hirokoji, Kamikyo-ku, Kyoto 602-8566, Japan; ^3^Department of Psychiatry, Kyoto Prefectural University of Medicine, Kawaramachi-Hirokoji, Kamikyo-ku, Kyoto 602-8566, Japan

## Abstract

We surveyed the attitudes of Japanese medical doctors toward complementary and alternative medicine (CAM) in 1999. It is supposed that the situation concerning CAM has been changing recently. The aim of the present study is to survey the attitude of doctors toward CAM again, and to examine changes in attitude over the last 6 years. The attitudes of medical doctors belonging to the Kyoto Medical Association toward CAM were surveyed by a structured, self-administered questionnaire in 1999 and 2005. The results showed that the doctors familiar with the term “CAM”, practicing CAM therapies, and attending meetings or training courses related with CAM, increased significantly from 1999 to 2005. The doctors who possessed knowledge of CAM also increased significantly from 1999 to 2005. Almost all doctors believed in the effectiveness of Kampo (Japanese traditional herbal medicine) and acupuncture. The number of doctors who believed in the effectiveness of aromatherapy and ayurveda increased significantly in 2005, compared with 1999. In the near future, 58% of doctors desired to practice CAM therapies. In conclusion, the numbers of doctors who practice CAM therapies, possess CAM knowledge and desire to practice such therapies have increased over the last 6 years in Japan.

## 1. Introduction

Complementary and alternative medicine (CAM) is defined by National Center for Complementary and Alternative Medicine in the United States (NCCAM) as a group of diverse medical and health-care systems, practices and products that are not presently considered to be part of conventional medicine.

CAM has been becoming more and more popular for a number of reasons, including dissatisfaction with issues related to conventional medicine, changes in attitude to the rights of patients and economical issues such as medical insurance systems [1–3].

There have been many studies on the attitude of medical doctors toward CAM practice; however, most of those studies have been conducted in Europe, North America and Oceania [4–8]. On the other hand, few studies have also been done in Asian countries [9].

Several Asian countries possess their own specific medical systems, which are occasionally practiced as conventional medicine in those countries. For example, in India, ayurveda is practiced on a nationwide scale, and in China, their specific traditional medical system including herbal medicine, acupuncture, acupressure, moxibustion, *t'ai chi ch'uan* and *qigong* is practiced in conjunction with modern Western medicine.

In Japan, Japanese traditional herbal medicine (Kampo), which had been originally introduced in the 5th to 6th century from China, has been greatly modified by adjustment by Japanese practitioners over a long time. Because Kampo was excluded from the authorized medical education and examination for the medical doctor's license by the government about 100 years ago, modern Western medicine is formally the conventional mainstream medicine in Japan at present. However, Kampo has been actually practiced by Japanese doctors. In fact, we surveyed the attitude of medical doctors toward CAM in 1999 [10, 11] and concluded as follows: Kampo (Japanese traditional medicine) was most frequently practiced, most commonly known and most highly trusted concerning its effectiveness by Japanese medical doctors. Furthermore, although Kampo may deeply influence beliefs concerning the effectiveness of other CAM therapies, Japanese doctors regard Kampo as somewhat independent of the other therapies.

It was also found in our previous survey that although the term “CAM” was recognized by only 45% of medical doctors, CAM was practiced by 73% of doctors, and the most common part of practiced CAM was Kampo.

Japanese medical insurance system covers Kampo, and partly acupuncture, moxibustion, massage and spa therapy, while it does not cover other CAM therapies. About 140 Kampo formulations and about 160 medicinal herbs are listed in the Japanese medical insurance system. Therefore, Japanese medical doctors can easily practice Kampo medicine, while they seldom practice acupuncture, moxibustion and massage, because these are practiced by acupuncturists, moxibustion practitioners and massagers.

In Japan, it is supposed that the situation concerning CAM has been undergoing a considerable change because many doctors and other medical professionals have become interested in CAM and a number of academic societies related to CAM have been established.

Therefore, we re-surveyed the attitude of medical doctors belonging to the same regional medical association toward CAM in 2005 and compared this with the results obtained in the 1999 survey.

## 2. Methods

### 2.1. Sample

The survey sample was drawn from the 1998 and 2003 Kyoto Medical Association (KMA) membership lists for surveys in 1999 and 2005, respectively. About 540 and 630 members were randomly selected from the 3774 and 4030 medical doctors of the KMA stratified by the district branch of the KMA in 1999 and 2005, respectively, because doctors may differ in attitudes among districts. The sampling fraction was one-seventh in all strata. The members surveyed in 1999 were excluded in the survey in 2005.

### 2.2. Data Collection

A structured, self-administered questionnaire was mailed to the randomly selected doctors, and was returned between the middle of February and end of April, both in 1999 and 2005, respectively.

The definition of CAM was informed to the doctors and the survey was performed. In the present study, CAM was defined as medicines other than the mainstream modern Western medicine, such as Kampo, acupuncture, moxibustion, dietary supplement, fasting, vegetarianism, herbal therapy, aromatherapy, spa therapy, chiropractic, yoga, quigong, art therapy hypnosis, imagery, thalassotherapy, flower therapy, prayer, homeopathy and ayurveda.

The questionnaires included a general profile such as age, year of graduation from medical school, specialty, working facility (private clinic, or hospital), practice of CAM, consultation by patients and referral to specialists, the degree of knowledge about CAM, and belief in the effectiveness of CAM and reasons, and also their desire to integrate CAM therapies into their practice in the near future and associated reasons. The last question was asked only in the survey of 2005.

Knowledge concerning the term “CAM”, knowledge about CAM, beliefs in the effectiveness of CAM and attitudes towards the integration of CAM into their practice in the near future were inquired by the single-choice method.

Reasons for their beliefs in the effectiveness, and reasons for their attitude towards CAM integration into their practice were inquired by the multiple-choice method.

### 2.3. Statistical Analysis

Proportions and means were examined by the *χ* ^2^-test using Statcel for Microsoft Excel 2003 for Windows (OMS, Saitama). When a *P* value was less than .05, it was considered to be significant.

## 3. Results

### 3.1. Response Rate

Replies were received from 364 and 405 medical doctors of the KMA in 1999 and 2005, respectively, which gave a response rate of 67% and 64%, with no significant difference.

### 3.2. General Profile

The respondents included 226 and 314 primary care doctors and 125 and 80 hospital doctors, ranging in age from those in their 20s to those in their 90s, with the median age being in the 50s. The average and standard deviation of ages were 57 ± 13 and 58 ± 12 in 1999 and 2005, respectively. The ratio of female to male doctors was 54/310 (15%) and 60/345 (15%). There were no significant differences in the age distribution and the ratio of females to males.

There were no significant differences in the general profiles (gender, age and working facilities) between respondents to this survey and non-respondents by the *X* ^2^ test (data not shown).

### 3.3. Changes in Attitudes toward CAM Therapies, Knowledge about Them and Beliefs in Their Effectiveness

We surveyed the attitudes of doctors toward CAM. In 1999, only 163 doctors (45%) were familiar with the term “CAM”, whereas 330 (82%) were familiar with it in 2005, which means there was a significant increase from 1999 to 2005 (*P* = 2.62 × 10^−25^) (Table 1).

Some form of CAM was practiced by 251/357 of the respondents (73%) in 1999 and 315/404 (78.0%) in 2005, which indicates a significant increase in the practice of CAM over the last 6 years (*P* = .0157).

The doctors who attended meetings or training courses related to CAM increased significantly from 1999 [103/354 (29%)] to 2005 [149/405 (37%)] (*P* = .0247).

The most common CAM practice was Kampo (70% of the respondents in 1999 and 78% in 2005), and the doctors practicing Kampo increased significantly from 1999 to 2005. In addition, 200 doctors (55%) in 1999 and 296 (73%) in 2005 were consulted by their patients regarding Kampo treatment, which also indicates a significant increase between 1999 and 2005.

Acupuncture and moxibustion were practiced by only 38 doctors (11%) in 1999 and by 34 (8%) in 2005, with no significant difference.

A small percentage of doctors practiced other forms of CAM therapy including chiropractic, aromatherapy, homeopathy, health spa therapy, ayurveda, hypnosis, flower therapy, thalassotherapy, herbal therapy, qigong, yoga, dietary supplement, imagery, meditation, art therapy and prayer in 1999, and this percentage increased significantly in 2005 (14%) compared with 1999 (8%) (*P* = .00722).

**Figure 1:**
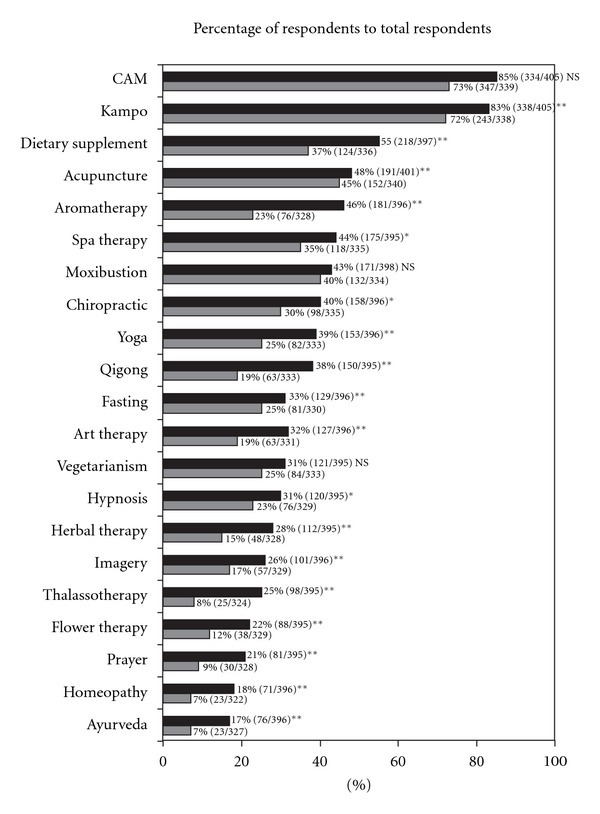
Changes in the knowledge of doctors about CAM between 1999 and 2005. The data obtained in 1999 and 2005 were analyzed by the *χ* ^2^-test. *P < .05; **P < .01; NS: not significant. Black indicates data for 2005 and grey for 1999.

Kampo was most commonly known to doctors (72% in 1999 and 83% in 2005), with a significant increase in 2005 compared with 1999 (Figure 1). Doctors who possessed knowledge of dietary supplement, aromatherapy, spa therapy, chiropractic, yoga, qigong, fasting, art therapy, hypnosis, herbal therapy, imagery, thalassotherapy, flower therapy, prayer, homeopathy and ayurveda increased significantly from 1999 to 2005.

**Figure 2:**
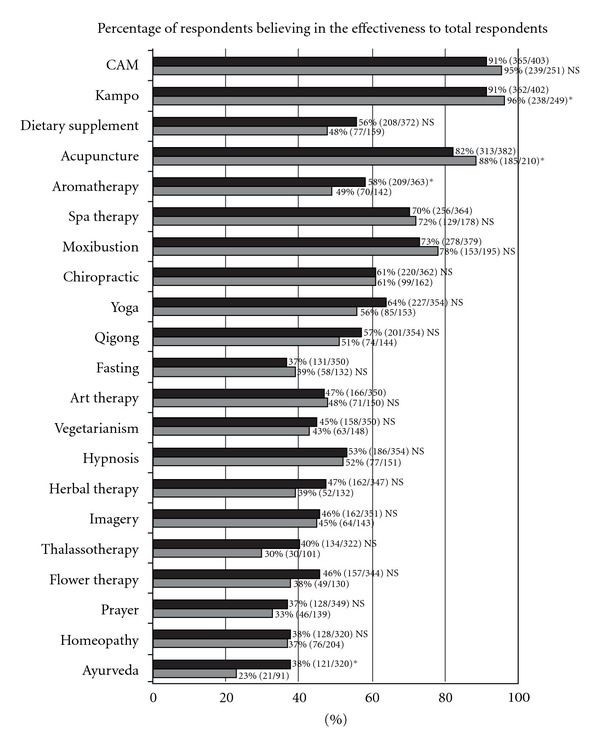
Changes in the beliefs of doctors concerning the effectiveness of CAM therapies between 1999 and 2005. The data obtained in 1999 and 2005 were analyzed by the *χ* ^2^-test. *P < .05; **P < .01; NS: not significant. Black indicates data for 2005 and grey for 1999.

Next, we surveyed doctors regarding their beliefs concerning the effectiveness of CAM therapies. The results showed that almost all doctors believed in the effectiveness of Kampo (96% and 91%, resp.) and acupuncture (88% and 82%, resp.) in 1999 and 2005. About 70% of doctors believed in the effectiveness of spa therapy, and more than 50% of doctors believed in the effectiveness of chiropractic, yoga and hypnosis. However, only a small percentage of doctors believed in the effectiveness of homeopathy, ayurveda, flower therapy, fasting and prayer (Figure 2).

Doctors who believed in the effectiveness of aromatherapy and ayurveda increased significantly in 2005 compared with 1999. However, beliefs in the effectiveness of Kampo and acupuncture decreased significantly in 2005 compared with 1999.

### 3.4. Changes in the Reasons for the Beliefs of Doctors Concerning the Effectiveness of CAM Therapies between 1999 and 2005

We compared the reasons for beliefs concerning the effectiveness of CAM therapies in 2005 with those in 1999 (Table 2). As for the item “Existence of reliable references, presentation in academic meetings, information about goods associated with CAM therapies and so on”, there were significant differences between 1999 and 2005. On the other hand, we also surveyed the reasons for the disbelief of doctors over the effectiveness of CAM therapies between 1999 and 2005 (Table 2). The results showed that the percentage of the items “no reliable information”, “unreliable data and conclusion in references” and “no reliable evidence” increased in 2005 compared with 1999.

### 3.5. Attitude of Doctors toward CAM Therapies in the Near Future

We asked doctors the question “Do you desire to integrate some form of CAM therapies into your practice in the near future?” only in the survey of 2005. The results showed that 58% of doctors desired to practice CAM therapies in the near future, whereas 42% did not (Table 3). The reasons for the desire to integrate CAM therapies were “limitation of modern Western medicine (62%)" and “desire of the patients to receive CAM (49%)”, whereas the reasons of doctors who did not desire to practice CAM therapies in the near future were “no solid scientific evidence (57%)", and “lack of knowledge and/or experience of CAM therapies by doctors themselves (69%)" (Table 4).

## 4. Discussion

Recently, interest in CAM has grown considerably in Japan. This is shown by the results of the present study. Namely, doctors familiar with the term “CAM” increased markedly in 2005 compared with those in 1999. And the doctors who practiced some form of CAM therapies and attended meetings or training courses related with CAM also increased significantly over the last 6 years (29%–37%), although these proportions are rather low, compared with the findings from a regional survey conducted by Berman et al. [12, 13].

Kampo medicine has been most commonly practiced by doctors (70% in 1999 and 78% in 2005). On the other hand, other CAM therapies have not been so commonly practiced (acupuncture: 11% in 1999 and 8% in 2005, other CAM therapies: 8% in 1999 and 14% in 2005). This means that Kampo medicine is a significant part of CAM in Japan.

Furthermore, as Kampo medicine has been taught to students in all Japanese Medical Schools since 2004, we anticipate that doctors who practice Kampo medicine will rapidly increase in the near future.

Doctors who possessed knowledge of CAM increased significantly in the 6 years from 1999 to 2005. Especially, Kampo medicine, dietary supplement, aromatherapy, spa therapy and chiropractic were well known by doctors. Moreover, the doctors possessing knowledge of yoga, qigong, fasting, art therapy, hypnosis, herbal therapy, imagery, thalassotherapy, flower therapy, prayer, homeopathy and ayurveda also increased significantly. This may be due to an increased interest of doctors in CAM therapies, and to the increased opportunity to be consulted by their patients or their family members.

As Eisenberg et al. [1] reported that the prevalence of CAM practice by citizens has been increasing worldwide. Medical professionals also practice CAM therapies in European and North American countries [14].

The prevalence of CAM practice is different depending on countries. For example, 51% of physicians in the United States practiced acupuncture [15], and vitamin therapy, herbal therapy, lifestyle diet, mineral therapy, and anti-oxidant therapy were frequently practiced by primary care physicians [7]. In the Netherlands, 51% of physicians practiced chiropractic therapy [16]. Furthermore, in Germany, 78% of physicians practiced herbal medicine, and 45% homeopathy [17].

The most popular CAM therapies that UK GPs referred their patients to were chiropractic treatment, acupuncture and osteopathy. German GPs referred their patients mainly to acupuncture treatment, chiropractic treatment and herbal medicine [5].

The situation concerning the kind of CAM therapies about which doctors possess knowledge is also very different from studies in American and European countries. For example, Reilly [18] reported that 86% of GPs had knowledge of hypnosis, 79%—acupuncture, 59%—homeopathy, 47%—osteopathy and only 19%—herbal medicine.

In Japan, Kampo is most commonly used, while in European and American countries, other CAM therapies are practiced. As discussed earlier, the situation of CAM therapies practiced is much different among countries. The reasons for the different situations are estimated to be dependent on the answers to the following questions:

- Are the CAM therapies covered by medical insurance systems?
- Are there reliable and officially authorized CAM therapy practitioners?
- Do medical doctors have knowledge of the CAM therapies and believe in their effectiveness?

As for the beliefs of doctors in the effectiveness of CAM therapies, there were no significant changes in almost all CAM therapies between 1999 and 2005, although the doctors who believed in the effectiveness of aromatherapy and ayurveda increased significantly in 2005 compared with 1999. The CAM therapies that doctors strongly believed were effective were Kampo and acupuncture, although beliefs in the effectiveness of Kampo and acupuncture decreased significantly in 2005 compared with 1999. We cannot understand the reasons for the significant decrease in the effectiveness.

Kurtz et al. [7] reported that American primary-care physicians believed that acupuncture, biofeedback, massage therapy and self-help groups were effective. These results were very different from ours except for the strong belief in the effectiveness of acupuncture. Similar results were stated in Leach's article [8]; namely, amongst 293 Dutch GPs, the majority did not believe in the effectiveness of many alternative therapies, particularly herbal medicine and nutritional supplementation. Nonetheless, over half of GPs believed that acupuncture, yoga and manual therapy were efficacious for various medical conditions.

The reasons for beliefs in the effectiveness of CAM therapies were the existence of reliable references, reliable presentation in academic meetings and reliable information about goods associated with CAM therapies, whereas the reasons for a lack of belief in the effectiveness were no reliable information, no reliable data and conclusions. This indicates that the factors associated with whether doctors believe in the effectiveness or not were dependent on the existence of reliable information or solid evidence.

Similar results were reported in other countries. Boutin et al. [4] pointed out that patients and physicians had similar interests in having CAM therapies provided, and both were hampered by a lack of information about many therapies. Schmidt et al. [5] also reported similar results in surveys in the United Kingdom and Germany.

Finally, we analyzed the attitude of doctors toward CAM in the near future, and the results showed that more than half of doctors desired to practice CAM therapies in the near future. Canadian physicians also desire to practice various CAM therapies at 15%–45%, depending on the kind of therapies [6].

The reasons why doctors desire to practice CAM in the near future, in the present study, were the limitations of Western medicine and desire of patients to receive them, while the reasons of doctors who did not desire to practice them were no solid evidence and lack of knowledge and experience of CAM therapies by doctors themselves. Solid evidence is important as a factor influencing doctors' attitudes. Therefore, solid scientific evidence of the effectiveness of CAM therapies should be established. Also in Germany, many academic doctors thought that the scientific evidence should be provided for the integration of CAM into the National Health System (NHS) [19].

In conclusion, the attitudes of Japanese doctors toward CAM were much different from those in American and European countries. The reasons for this may be due to the unique position of Kampo in CAM of Japan [10]. Namely, Kampo is considered to be the useful alternative medicine widely practiced in Japan, and gains an advantage over many other CAM therapies. This situation is similar to those in many Asian countries including China, India, Korea and Vietnam.

## Figures and Tables

**Table 1:** Changes in attitudes of doctors toward CAM therapies between 1999 and 2005.

	2005	1999	*P*-value
	Yes/total (%)	Yes/total (%)	
General			

Practice CAM	323/404 (80)	267/364 (73)	*
Familiar with the term “CAM”	330/404 (82)	163/359 (45)	**
Attend meetings or training courses	149/405 (37)	103/354 (29)	*

Kampo			

Practice	315/404 (78)	251/357 (70)	*
Consulted by patients	296/404 (73)	200/361 (55)	**
Refer patients to specialists	54/404 (13)	44/362 (12)	NS

Acupuncture and Moxibustion			

Practice	34/404 (8)	38/362 (11)	NS
Consulted by patients	200/404 (49)	156/362 (43)	NS
Refer patients to specialists	81/404 (20)	79/363 (22)	NS
Other CAM therapies	57/405 (14)	28/354 (8)	**

By *χ* ^2^-test; *P < .05; **P < .01.

NS, not significant.

**Table 2:** Changes in the reasons for the beliefs and disbelief of the doctors concerning the effectiveness of CAM therapies between 1999 and 2005.

	2005	1999	*P*-value
Reasons for the belief			
(1) Improvement of patients during treatment with CAM	176 (56%)	147 (56%)	NS
(2) Experience of the improvement by	91 (29%)	65 (25%)	NS
themselves			
(3) Existence of reliable references, presentation	146 (46%)	104 (40%)	*
in academic meetings, information about			
goods associated with			
CAM and so on			
(4) Recommendation by reliable persons	16 (5%)	9 (3%)	NS
(5) Others	61 (19%)	41 (16%)	NS
Respondents	315 (100%)	261 (100%)	
No answer	90	103	

Total	405	364	

Reasons for the disbelief			
(1) No reliable information	154 (69%)	111 (59%)	*
(2) Unreliable data and	80 (36%)	46 (25%)	**
conclusion in references			
(3) No reliable evidence	165 (74%)	94 (50%)	**
(4) No experience of cases in which CAM was effective	30 (14%)	29 (56%)	NS
(5) Experience of	76 (34%)	55 (29%)	NS
aggravation, delay in			
treating, side effects in			
patients treated with			
CAM			
(6) No experience of the improvement by	13 (6%)	12 (6%)	NS
themselves			
(7) Others	33 (15%)	19 (22%)	NS
Respondents	222 (100%)	187 (100%)	
No answer	183	177	

Total	405	364	

By *χ* ^2^-test; *P < .05.

NS, not significant.

**Table 3:** Desire to practice CAM therapies in the near future.

(1) Practice at present, also in the near future	176 (45%)
(2) Not practice at present, but in the future	51 (13%)
(3) Practice at present, but not in the near future	9 (2%)
(4) Neither practice at present nor in the near future	159 (40%)

Total	395 (100%)

No answer	2
Invalid answer	9

By *χ* ^2^-test; *P < .05; **P < .01.

NS, not significant.

**Table 4:** The reasons of doctors who desire or do not desire to practice CAM therapies in the near future (by multiple choice method).

	
Reasons for desiring	
(1) Solid scientific evidence	51 (22%)
(2) Limitation of Western medicine	141 (62%)
(3) Desire of patients to receive CAM	110 (49%)
(4) Others	42 (19%)

Total	227 (100%)

Invalid answer	25
Reasons for not desiring	
(1) No solid scientific evidence	95 (57%)
(2) Lack of knowledge and/or experience of doctors themselves	116 (69%)
(3) No demands of the patients to receive CAM	33 (20%)
(4) Problems of medical insurance systems specific to Japan	37 (22%)
(5) Others	13 (8%)

Total	168 (100%)

Invalid answer	67

By *χ* ^2^-test; *P < .05; **P < .01.

NS, not significant.
